# Surface plasmon resonance based biosensor: A new platform for rapid diagnosis of livestock diseases

**DOI:** 10.14202/vetworld.2016.1338-1342

**Published:** 2016-12-01

**Authors:** Pravas Ranjan Sahoo, Parthasarathi Swain, Sudhanshu Mohan Nayak, Sudam Bag, Smruti Ranjan Mishra

**Affiliations:** 1 Department of Veterinary Biochemistry, Orissa University of Agriculture & Technology, Bhubaneswar, Odisha, India; 2 Department of Livestock Production and Management, Orissa University of Agriculture & Technology, Bhubaneswar, Odisha, India; 3 Department of Clinical Medicine, Orissa University of Agriculture & Technology, Bhubaneswar, Odisha, India; 4 National Institute of Animal Health, Baghpat, Uttar Pradesh, India; 5 Department of Veterinary Physiology, Orissa University of Agriculture & Technology, Bhubaneswar, Odisha, India

**Keywords:** biosensor, livestock sector, surface plasmon resonance

## Abstract

Surface plasmon resonance (SPR) based biosensors are the most advanced and developed optical label-free biosensor technique used for powerful detection with vast applications in environmental protection, biotechnology, medical diagnostics, drug screening, food safety, and security as well in livestock sector. The livestock sector which contributes the largest economy of India, harbors many bacterial, viral, and fungal diseases impacting a great loss to the production and productive potential which is a major concern in both small and large ruminants. Hence, an accurate, sensitive, and rapid diagnosis is required for prevention of these above-mentioned diseases. SPR based biosensor assay may fulfill the above characteristics which lead to a greater platform for rapid diagnosis of different livestock diseases. Hence, this review may give a detail idea about the principle, recent development of SPR based biosensor techniques and its application in livestock sector.

## Introduction

The India’s livestock sector is an important sector which contributes the largest economy of the country. However, the occurrence of most of the viral, bacterial, and fungal diseases, which impacts a great economy loss to India, is now a major concern in both small and large ruminants [1]. Hence, an accurate, rapid diagnosis is required to control the economic loss by employing a good control strategy. However, the current diagnostic methods available for detecting animal diseases are expensive, time consuming and laborious which implies the researcher to develop a rapid, less expensive, and very sensitive diagnostic method. The surface plasmon resonance (SPR) based biosensor assay may fulfill the above parameters, can be extensively used for rapid diagnosis of livestock disease. A biosensor is label free analytical device which is used for the detection of an analyte, mainly consists of two components, i.e., biological component and physicochemical detector [2]. The biological component, i.e., tissue, microorganisms, organelles, cell receptors, enzymes, antibodies, and nucleic acids interact with the analate producing a signal which is transformed by the physicochemical detector (transducing element) to another signal that can be easily measured and quantified [3]. The transducer detects the interaction between the target analyte and the immobilized biological materials, producing physicochemical, i.e., optical, piezoelectric, electrochemical, thermal, acoustic, and magnetic signal. It has been demonstrated that SPR based biosensor may be used as an exceedingly powerful and quantitative probe of the interactions of a variety of biopolymers with various ligands, biopolymers, and membranes including protein: Ligand, protein: Protein, protein: DNA and protein: Membrane binding which provides a means not only for identifying these interactions and quantifying their equilibrium constants, kinetic constants, and underlying the energetics but also for employing them in very sensitive, label free biochemical assays for diagnosis of different emerging livestock diseases. Hence, this article will highlight the basic principle and design of this assay which would provide a good platform for reviewers to understand about the methodology and application of SPR based biosensor in livestock sector.

## SPR

It is one of the important surface phenomena behind many color based biosensor and different lab-on-a-chip sensors for measuring adsorption of materials onto planar metal (typically gold or silver) surfaces or onto the surface of metal nanoparticles (NPs) [4]. SPR is nothing but one of the resonant oscillation of conduction electrons at the interface between a negative and positive permittivity material stimulated by incident light [5], and these oscillations are very sensitive to any change of this boundary, therefore, can be used as a sensing method for detection of external medium.

### Bulk SPR and localized SPR (LSPR)

Now physicists, chemists and materials scientists and biologists use the concept of excitation of SPs by light as a bulk SPR or SPR in terms of conventional usage for planar surfaces and LSPR for metallic NPs or metallic nanostructures [6]. In bulk SPR case, the momentum of the SP mode, kSP, being greater than that of a free space photon of the same is resulted after the interaction between the surface charge density and the electromagnetic field. Two main techniques are involved to measure this missing momentum one is attenuated total reflection method which utilizing prism coupler to enhance the momentum of the incident light and the others are metallic grating method based on a periodic corrugation in the metallic surface [7].

In LSPR case, the LSPs are generated by scattering the light from a topological defect on the surface, such as a metallic nanostructures or NPs [8]. Plasmon that oscillates locally around the NP is formed after interaction of light with metallic particles or nanostructures with much smaller than the incident wavelength. Dipole plasmon resonance and quadruple plasmon resonance are observed in smaller spherical NPs, e.g., Au or Ag, with diameters <30 nm, and in larger particles, respectively. The magnitude, peak wavelength and spectral bandwidth of the plasmon resonance associated with an NP are dependent on the particle’s size, shape, material composition, as well as its local dielectric environment [2].

### SPR-based biosensor

**Figure-1:**
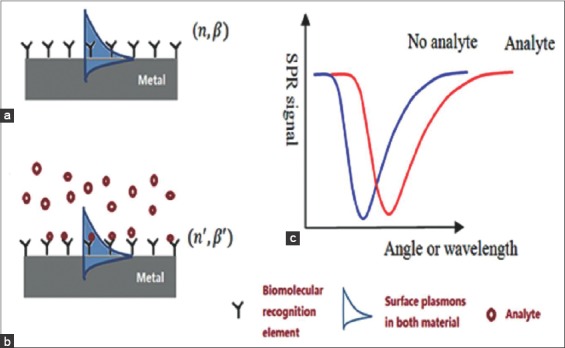
(a-c) Surface plasmon resonance biosensing principle [2].

A local increase in the refractive index at the metallic surface is produced after recognition and capturing of target analyte with the bimolecular recognition elements on the surface of metal [9]. This refractive index change may increase the propagation of constant of SPs along the metallic surface which can be accurately measured by different optical means such as intensity modulation, angular modulation, wavelength modulation, phase modulation, and even polarization modulation. There is transfer of energy from incident photons to SPs due to this excitation of SPs at metal-dielectric interface. A sharp dip is observed at resonance angle if a graph is plotted between reflection intensity (SPR signal) with an incident angle (Figure-1). Hence, this correlation between refractive index changes on the metallic surface and the spectral shift of the resonance dip is employed in the biosensor application.

### Biorecognition element

This is the valuable component of the biosensor for its specificity. Various types of biorecognition elements, i.e., antibodies, peptides, and aptamers are available for specific detection of analyte [10]. Although conventional polyclonal and monoclonal antibodies are available, recombinant antibodies consisting of genetically manipulated fused antigen binding domains of common antibodies are another source for biosensor [11]. The immobilization of these biorecognition elements on the gold surface is one of the important tasks for better performance of the biosensor. This immobilization is mainly done by either physical adsorption method [12], i.e., weak electrostatic interaction or by stable covalent attachment through exposing functional groups of accessible amino acids, e.g., lysine (amine group), cysteine (thiol group), with suitable types of derivatized surfaces, e.g., carboxylic acid, aldehyde, maleimide, and vinyl sulfone. Different site-specific immobilization techniques, e.g., Diels-Alder cycloaddition [13], “click” chemistry [14], and peptide ligation [15] are employed nowadays in the better performance of biosensor. The site-specific immobilization is also done by utilizing different biochemical affinity reaction, e.g., biotin-avidin/streptavidin reaction [16], histidine cheated metal ion interaction [17], or DNA hybridization [18]. In SPR biosensors, peptides are now extensively used as biorecognition materials for the study of protein structure and function [19] and also used for the detection of heavy metals and small ligand [20]. Immobilization of peptides to the metallic surface is done by electrostatic attraction and amine- or thiol-based covalent coupling. Aptamers are nucleic acid ligands (RNA, ssDNA, modified ssDNA, or modified RNA) are used against a range of targets including drugs, proteins, and even supramolecular complexes such as viruses or bacteria [21]. A highly effective recognition surface, i.e., increased density of active surface groups is produced by the technique of self-assembled monolayers; kind of ordered molecular assemblies of different organic materials are used to separate the immobilized molecules with a minimal distance [22].

### Different Measurement Formats for SPR Biosensor

Three important measurements formats, i.e., direct detection [23], sandwich [24], and competitive inhibition assay [25] have been developed in SPR biosensor ensuring a measurable sensor response. Several kinds of metallic NPs including Au NPs [26], Pd NPs [27], and Pt NPs [28] had been applied to increase the SPR sensitivity for detecting all kinds of biomolecules, for example, the formation of antigen-antibody complexes, DNA hybridization [29], formation of aptamer substrate complexes [30], or enzymatic transformations [31].

### Sensing platforms for the biosensor

It is the important component of biosensor on which there is reaction between target analytes with biomolecular material. In this regards, SPR transducer is often used in conjunction with microfluidic flow channels, which allows use of small volumes of expensive reagents [32] and improves the sensor performance for multiple analyte analysis.

### Advances of SPR Based Biosensor Assay in Livestock Sector

Different molecular techniques have been existing for diagnosis of livestock diseases, but all these assays are time-consuming and not that much sensitive. To fulfill this lacuna, there was some advancement of SPR based biosensor assay for rapid diagnosis of different livestock diseases summarizing in Table-1. A sensitive and label-free analytical approach for the detection of porcine circovirus Type 2 (PCV2) instead of PCV2 antibody in serum sample was systematically investigated based on SPR with an establishment of special molecular identification membrane [33]. To prevent the respiratory infection caused by feline calicivirus (FCV), an antibody based assay was performed by first immobilizing anti-FCV to an SPR chip surface for detection of this virus [34]. For rapid diagnosis of Classical Swine Fever in pig, the biomolecular interaction between the virus and the serum antibody was investigated by Biacore SPR system on relative time [35]. *Escherichia coli* in the large animal was diagnosed by regular grating coupled SPR with direct format assay with a detection limit of 50 cfu/ml [36]. The rabies virus in livestock can be detected by linking N protein specific antibody on one CM5 chip based on SPR [37]. A new SPR biosensor based on an array format, which allowed immobilizing nine tuberculosis (TB) antigens onto the sensor chip, was constructed for simultaneous determination of multiple TB antibodies in serum [38] in the case of tuberculosis. It has been reported that simultaneous and specific detection of *Salmonella* serovars in animal was diagnosed by this assay [39]. Multiple sclerosis was detected by glycogen based SPR assay in animal [40], and finally, it has been reported that in the case of mad cow disease, this SPR based assay was used to develop a rapid, label-free and sensitive immunoassay for detection of prion protein [41].

**Table-1:** Different formats of SPR assay developed against various livestock disease.

Name of disease/microorganism	Ligand attached to SPR chip	References
PCV	PCV2 antibody	Jiang *et al*. [33]
FCV	anti-FCV	Yakes *et al*. [34]
CSF	CSF virus	Mustafa *et al*. [35]
*E. coli*	Anti *E. coli* Ab	Wang *et al*. [36]
Rabies	N protein specific antibody	Xu *et al*. [37]
TB	TB antigen	Hsieh *et al*. [38]
Mad cow disease	Anti PrP monoclonal Ab	Jiayu *et al*. [41]

PCV=Porcine circovirus, CSF=Classical Swine Fever, TB=Tuberculosis, FCV=Feline calicivirus, SPR=Surface plasmon resonance, *E. coli*=*Escherichia coli*

## Conclusion

It can be concluded that the SPR based biosensor assay may provide a label free rapid platform for diagnosis of infectious and noninfectious diseases of livestock. This assay can be used as alternative cheapest diagnostic tools in clinical areas, particularly in the developing countries. It has not only widened avenues in therapeutic measures but also in research based screening of many infectious livestock diseases. This assay could test a large number of samples in quick time which can lead to quick control and prophylactic strategies against various important livestock diseases.

## Authors’ Contributions

Each and every author has contributed the relevant literature in preparation of this work of review. PRS carried out his investigations and experimentations on the mentioned topic. PS and SRM searched various related topics for better reference purpose. SMN corrected the grammatical errors exists in the manuscript and SB designed the proper format of the manuscript. All authors read and approved the final manuscript.
